# Alternate day versus daily oral iron for treatment of iron deficiency anemia: a randomized controlled trial

**DOI:** 10.1038/s41598-023-29034-9

**Published:** 2023-02-01

**Authors:** Elamparithi Pasupathy, Ravichandran Kandasamy, Kurien Thomas, Aneesh Basheer

**Affiliations:** 1 Department of General Medicine, Sri Venkateshwaraa Medical College and Research Centre, Pondicherry, India; 2 Department of Biostatistics, Pondicherry Institute of Medical Sciences, Pondicherry, India; 3 Department of General Medicine, Pondicherry Institute of Medical Sciences, Pondicherry, India; 4 Department of General Medicine, DM Wayanad Institute of Medical Sciences, Naseera Nagar, Wayanad, Kerala 673577 India

**Keywords:** Diseases, Health care

## Abstract

Oral iron is the mainstay of treating iron deficiency anemia. Recent studies indicate better fractional iron absorption with alternate day supplementation. However, the optimal supplementation strategy is unclear. We compared effectiveness of daily versus alternate day supplementation of oral iron for treatment of iron deficiency anemia. This double blind, active control, randomized controlled trial was conducted on two hundred adults having hemoglobin 10 g/dL or less with microcytic hypochromic anemia and/or serum ferritin below 50 ng/mL. They were randomized to receive either two Ferrous sulfate tablets containing 60 mg elemental iron (120 mg total) on alternate days or single tablet of 60 mg elemental iron daily for 8 weeks. Primary outcome was mean change in hemoglobin at week 8 from baseline. Mean hemoglobin was 6.53 (± 1.89) and 6.68 (± 1.89) g/dL in the alternate day and daily arms respectively. Mean change in hemoglobin was + 1.05 ± 1.34 g/dL in alternate day arm and + 1.36 ± 1.51 g/dL in daily arm (*p* = 0.47) at week 8. There were no statistically significant differences between the arms with respect to any secondary outcome. There is no significant difference between alternate day and daily iron administration in improving hemoglobin. Randomized controlled trials enrolling more participants for longer periods of supplementation and evaluating clinically relevant outcomes like change in hemoglobin may be useful in identifying the ideal dosing strategy.

**Trial Registration**: Clinical Trial Registry of India (CTRI/2019/01/017169).

## Introduction

Iron deficiency is the most common cause of anemia in developing nations. The National family health survey of India reported that 32.4% and 2.2% of women had mild and severe anemia respectively^1^. Oral iron therapy is an inexpensive and effective means of replenishing iron stores in iron deficiency anemia^2^. A pooled analysis of randomized controlled trials revealed that more than 60% of individuals who received oral iron had an increase in serum hemoglobin by the end of second week^3^. However, effectiveness of oral iron is hampered by poor absorption and gastrointestinal side effects such as metallic taste, constipation, diarrhea, and epigastric discomfort. Non-adherence to daily iron has been estimated to range from 10 to 32%^4,5^. Stoffel et al.^6^ showed that daily doses of oral iron reduced its absorption by increasing serum hepcidin levels. This study also found that administration of oral iron on alternate days as single doses improved iron absorption^6^. Theoretically, using lower doses and increasing time interval between consecutive doses might reduce the amount of unabsorbed iron in the gastrointestinal tract resulting in lesser gastrointestinal side effects. However, the major concern with alternate day dosing is that only half the total amount of iron is supplemented per unit time compared to daily dosing. We aimed to test the hypothesis that alternate day oral iron as single dose is superior to daily single dose of oral iron in improving anemia.

## Methods

### Participants

This was a prospective, parallel arm, double blind, active control randomized controlled trial conducted at a tertiary care teaching hospital in south India between January 2019 and December 2020. The primary objective of the study was to determine the efficacy and safety of alternate day oral iron therapy compared to daily oral iron in participants with iron deficiency anemia. Two hundred adults aged 16 years and above with hemoglobin of 10 g/dL or less with microcytic hypochromic anemia on peripheral smear and/or serum ferritin below 50 ng/mL were included in this study. Individuals with chronic kidney disease, chronic liver disease, cardiac failure, concomitant malignancy, hemolytic anemias (including hemoglobinopathies) and pregnancy were excluded from the study. Patients who received blood transfusions within past 3 months and/or suffering from severe anemia requiring transfusion were also excluded.

### Randomization and interventions

Following written informed consent, eligible participants were randomized to the alternate arm or daily arm by computer generated sequence of random numbers. Block randomization with a block size of four was used. Allocation of study medication was concealed using sequentially numbered opaque sealed envelopes. This sequence was unknown to the investigators. The random sequence was generated by the statistician who was not directly involved in the care of patients while participants were enrolled by the principal investigator after determining eligibility. Eligible participants were then assigned interventions by a clinician who was not part of the trial. The alternate arm participants received two Ferrous sulphate tablets (100 mg each) containing 60 mg elemental iron (total 120 mg) as a single morning dose on alternate days for a period of 2 months. The daily arm participants received one Ferrous sulphate tablet containing 60 mg elemental iron and one placebo tablet (total 2 tablets) daily for 2 months. In order to blind study participants, the alternate arm received placebo every other day similar in appearance to the iron tablets. All participants were followed up at 2, 4 and 8 weeks. Clinical examination, enquiry about side effects and blood investigations for hemoglobin, serum ferritin and reticulocyte count were carried out at each visit. The primary outcome was mean change in hemoglobin from baseline at week 8. Secondary outcomes included proportion of participants who achieved a rise of 2 g/dL or more in hemoglobin at week 8 compared to baseline, change in mean reticulocyte count at 2 weeks, change in mean serum ferritin levels at week 4 from baseline and adverse events.

### Statistical analysis and sample size

Intention to treat analysis were performed for all primary and secondary outcomes. We represented continuous variables as means with standard deviations, and risk estimates as proportions. Mean hemoglobin between groups and within groups were compared using repeated measures ANOVA followed by post hoc test for within groups. Comparison of proportions between groups was determined by Chi-square/Fishers exact test. Changes in biochemical parameters from baseline were compared using Mann–Whitney Test. *p* values less than 0.05 were considered statistically significant. Sample size was calculated as 200 participants assuming a mean change of 2 g/dL (and standard deviation of 5) at 8 weeks between the alternate day and daily arms, a power of 80% and alpha error of 5%.

### Ethical approval

All study related procedures were carried out in accordance with Good Clinical Practice guidelines. Written informed consent was obtained from all participants and/or their legal guardians. The study was approved by the Institutional Ethics committee (IEC:RC/18/71) of Pondicherry Institute of Medical Sciences and registered with the Clinical Trial Registry of India (CTRI/2019/01/017169; first registered on 21/01/2019).

## Results

**Figure 1:**
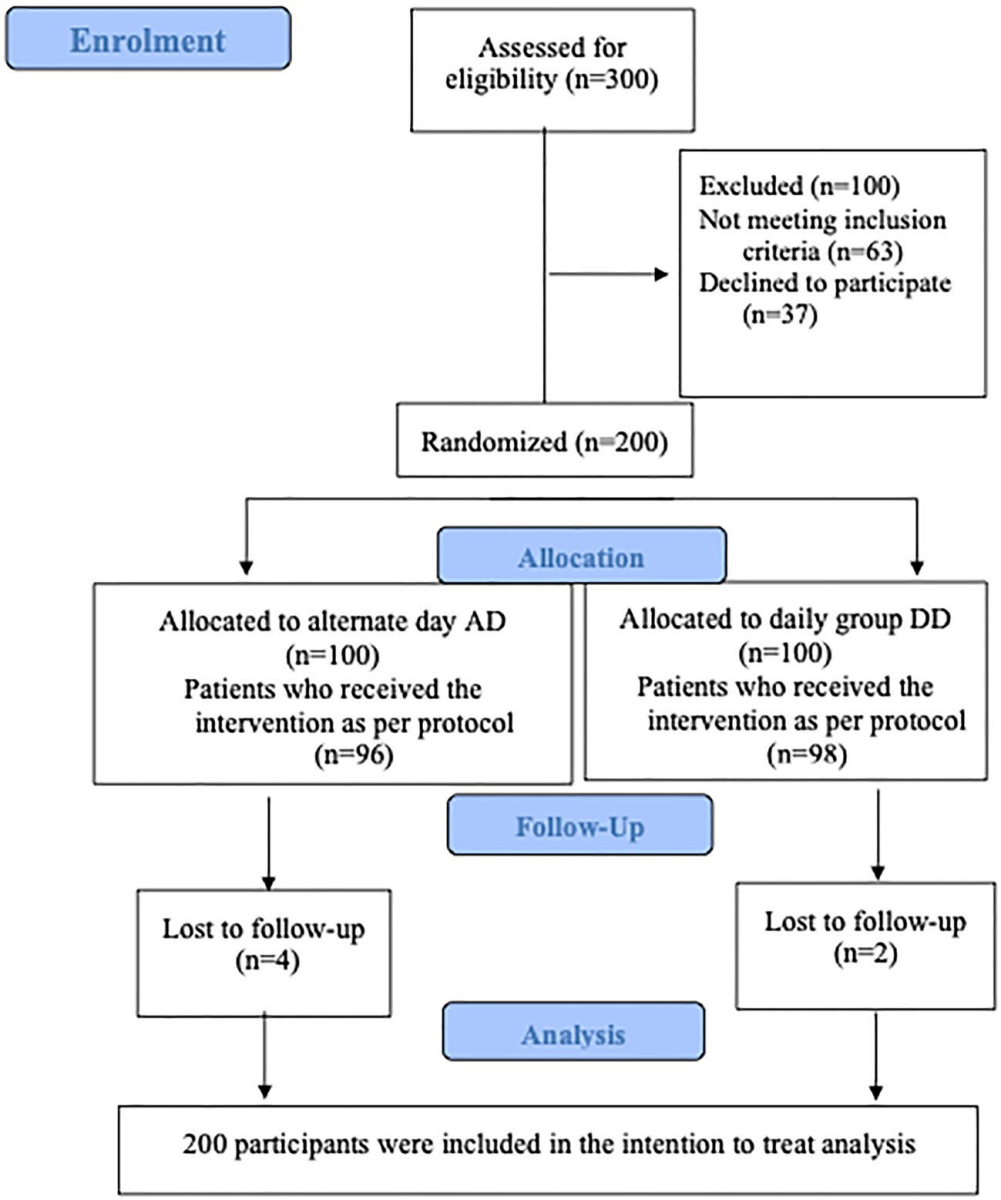
CONSORT flow diagram depicting participant screening, recruitment, randomization and follow up.

Three hundred participants who attended the outpatient and inpatient units of the hospital were screened for eligibility between January 2019 and December 2020. After excluding ineligible participants, we randomized 200 participants to the alternate day and daily arms (Fig. 1).

Baseline characteristics of participants in the two arms were similar (Table 1). Mean hemoglobin was 6.53 (± 1.89) g/dL and 6.68 (± 1.89) g/dL in the alternate day and daily arms respectively. Overall, mean serum ferritin was 63.89 (± 219.43) ng/mL.

**Table 1:** Baseline characteristics of participants randomized to the alternate and daily dosing arms.

Characteristics	Alternate day group (n = 100)	Daily dose Group (n = 100)	Total
Age (years)	49.23 (± 15.71)	45.33 (± 17.31)	47.28 (± 16.60)
Hemoglobin (g/dl)	6.53 (± 1.89)	6.68 (± 1.89)	6.60 (± 1.88)
Packed cell volume (%)	21.28 (± 5.16)	21.65 (± 5.25)	21.47 (± 5.20)
Total WBC counts (cells/microL)	7568 (± 3584.39)	7478 (± 3138.05)	7523 (± 3360.45)
MCV (fL)	62.29 (± 8.83)	62.01 (± 8.95)	62.15 (± 8.87)
MCH (pg)	18.89 (± 3.92)	19.06 (± 3.86)	18.97 (± 3.88)
MCHC (%)	30.03 (± 2.48)	30.16 (± 2.28)	30.09(± 2.38)
RCW (%)	23.79 (± 4.07)	23.80 (± 3.97)	23.80 (± 4.01)
Platelet counts (cells/microL)	299,620 (± 142,764.25)	311,130 (± 130,666.02)	305,375 (136,626.49)
Ferritin (ng/ml)	56.86 (± 192.97)	70.92 (± 243.82)	63.89 (± 219.43)
Reticulocyte count (%)	0.74 (± 0.50)	0.78 (± 0.47)	0.76 (± 0.49)
ESR (mm/hr)	27.56 (± 24.14)	29.59 (± 26.93)	28.57 (± 25.53)
Total Bilirubin(mg/dl)	0.42 (± 0.27)	0.45 (± 0.34)	0.44 (± 0.30)
SGOT (U/L)	29.07 (± 27.81)	39.31 (± 122.24)	34.19 (± 88.57)
SGPT (U/L)	20.88 (± 17.80)	25.02 (± 45.46)	22.95 (± 34.50)
Alkaline phosphatase (U/L)	87.3 (± 26.89)	97.52 (± 70.22)	92.41 (± 53.28)
Total protein (g/dl)	6.69 (± 0.70)	6.58 (± 0.66)	6.63 (± 0.68)
Serum Albumin (g/dl)	3.76 (± 0.55)	3.73 (± 0.53)	3.75 (± 0.54)
Urea (mg/dl)	21.88 (± 12.56)	20.72 (± 9.97)	21.30 (± 11.32)
Serum Creatinine (mg/dl)	0.70 (± 0.24)	0.67 (± 0.24)	0.68 (± 0.24)

**Figure 2:**
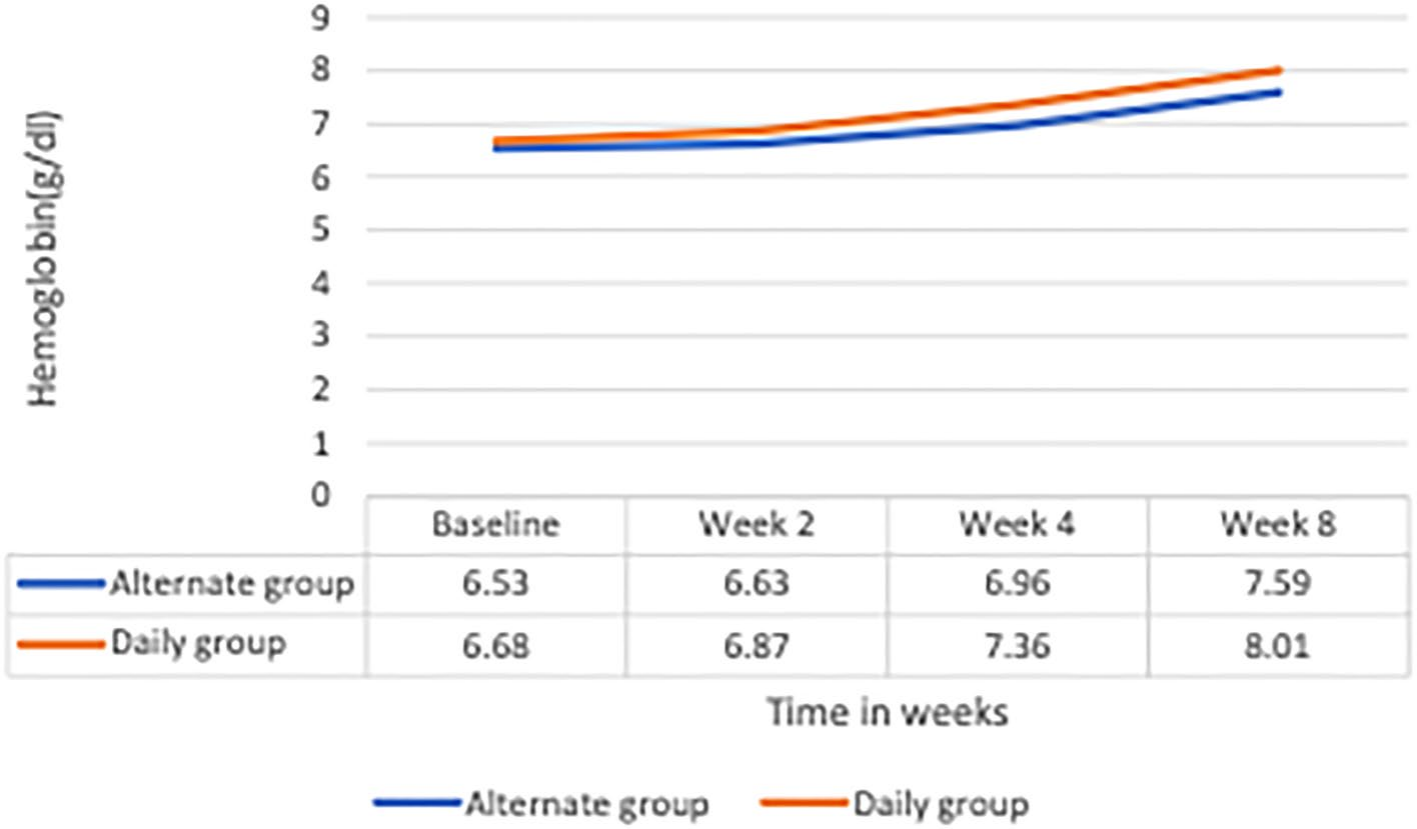
Change in mean hemoglobin levels at weeks 2, 4 and 8 from baseline in patients randomized to daily and alternate day iron therapy.

The mean hemoglobin rose to 7.59 (± 1.58) g/dL in the alternate day arm compared to 8.01 (± 1.80) g/dL in the daily arm at the end of 8 weeks. While hemoglobin increased significantly at week 8 from baseline in both the arms, the mean of change in hemoglobin from baseline was + 1.05 ± 1.34 g/dL in alternate day arm and + 1.36 ± 1.51 g/dL in the daily arm (*p* = 0.47). Figure 2 depicts the comparison of hemoglobin increments at 2, 4 and 8 week between the two arms. Results of repeated measures ANOVA indicated no difference in the mean hemoglobin value between groups (*p* = 0.226). However, there was significant increase in hemoglobin value from baseline to 2nd, 4th and 8th week, in both the arms (*p* < 0.001). More specifically, significant increase in hemoglobin value at 8th week from 4th week and 4th week from 2nd week was observed in both the arms (*p* < 0.001). But significant increase in hemoglobin was observed between baseline and 2nd week only in daily dose group (*p* = 0.002) and not in alternate day dose group (*p* = 0.157). The interaction, as seen in Fig. 2, was not statistically significant (*p* = 0.138). The mean of change in reticulocyte count at week 2 from baseline was 0.36 in both arms (*p* = 0.97). There were no statistically significant differences between the two arms with respect to any of the other secondary outcomes (Table 2). None of the participants discontinued medication due to adverse effects. Among the adverse events, nausea alone was reported more among the alternate day arm participants compared to daily arm participants (10.3% vs. 2%; *p* = 0.015) at week 4 (Table 3).

**Table 2:** Major secondary outcomes in the two arms at weeks 2, 4 and 8 of randomization.

Outcome	Alternate group	Daily group	*P* value
Change in retic count at week 2 compared to baseline	0.36 ± 0.44	0.36 ± 0.42	0.97
Change in ferritin at week 4 compared to baseline	30.84 ± 159.02 ng/ml	17.69 ± 40.26 ng/ml	0.32
Proportion of participants with at least 2 g/dL rise in Hb at week 8 compared to baseline	17 (17.71%)	22 (22.45%)	0.41

**Table 3:** Adverse events among participants randomized to daily and alternate day iron therapy.

	Alternateday group (n = 100)	Daily group (n = 100)	*P* value
Week 2			
Nausea	10 (10.0)	11 (11.0)	0.818
Vomiting	14 (14.0)	14 (14.0)	1.000
Gastric discomfort	9 (9.0)	8 (8.0)	0.800
Altered bowel habits	4 (4.0)	9 (9.0)	0.152
Metallic taste	6 (6.0)	7 (7.0)	0.774
Week 4			
Nausea	10 (10.3)	2 (2.0)	**0.015**
Vomiting	11 (11.3)	7 (7.0)	0.291
Gastric discomfort	11 (11.3)	13 (13.0)	0.722
Altered bowel habits	9 (9.3)	5 (5.0)	0.243
Metallic taste	4 (4.1)	3 (3.0)	0.718
Week 8			
Nausea	6 (6.2)	4 (4.1)	0.535
Vomiting	5 (5.2)	4 (4.1)	0.746
Gastric discomfort	3 (3.1)	6 (6.1)	0.498
Altered bowel habits	3 (3.1)	2 (2.0)	0.681
Metallic taste	2 (2.1)	4 (4.1)	0.683

Significant values are in bold.

## Discussion

This randomized controlled trial of 200 participants with iron deficiency anemia with hemoglobin of 10 g/dL or less found that there were no differences between alternate day oral iron supplementation compared to daily supplementation in terms of increase in hemoglobin, serum ferritin or reticulocyte count at any of the prespecified follow up points. No major differences were noted between the two arms with regard to adverse effects, except for more nausea in the alternate day arm at week 4.

Early reports of potential benefit from intermittent versus consecutive iron supplementation come from a Cochrane systematic review of randomized controlled trials comparing the two strategies on pregnant women^7^. This and other studies on menstruating females have demonstrated that intermittent administration of oral iron may be better than daily iron^8^. Moretti et al. performed a study based on the recognition that iron supplementation causes rise in hepcidin levels that could impede absorption of further doses of iron. Their investigation of 54 non-anemic but iron-depleted women demonstrated that lower dosages ranging between 40 and 80 mg or elemental iron, and avoidance of twice daily doses improved fractional absorption of iron^9^. Hence, the authors suggested that alternate day iron supplementation might be advantageous. However, sample size was small; duration of iron supplementation was very short (2 days), and the participants did not have anemia.

In a subsequent study, Stoffel et al. determined the iron absorption from oral iron administered on consecutive versus alternate days^6^. Participants of this study were iron deficient women who received 60 mg elemental iron for 14 days consecutively or on alternate days for 28 days. As expected, serum hepcidin was higher in the consecutive day group compared to alternate day group. The mean cumulative fractional iron absorption was 16.3% in the consecutive day group compared to 21.8% in alternate day group (*p* = 0.001). In the same study, the authors assigned ten women to receive once-daily dose and ten women to receive two divided doses. While no differences were observed between these two groups of women in terms of fractional iron absorption or total iron absorption, twice daily administration caused hepcidin to rise more than once daily regimen^6^. These studies also did not enroll anemic patients. Further, small sample size and short duration of supplementation limited extrapolation of findings to real world settings. Another limitation of these studies was the lack of clinically relevant outcomes such as change in hemoglobin.

In 2020, Stoffel and colleagues studied the effect of alternate versus daily iron supplementation in iron-deficient and anemic women^10^. Doses of 100 mg or 200 mg iron were given in a cross over design to 19 women. Supplementation was only for 3 doses with a wash out period of 16 days. The fractional iron absorption was significantly higher in the alternate day dosing group compared to consecutive day group. Although the inclusion of participants with anemia was a strength, the median hemoglobin was 11.5 g/dL, much higher than values seen in clinical practice in developing countries where iron deficiency anemia is a major problem.

Another study on anemic infants from Kenya reiterated the findings of Stoffel in adults, although the decrease in iron absorption from consecutive day dosing was modest compared to alternate day dosing^11^.

While most studies have used iron absorption as the primary outcome, Kaundal et al.^12^ reported the results of the first randomized controlled trial using improvement in hemoglobin as the primary outcome. This trial assigned 62 participants with iron deficiency anemia to receive either 60 mg elemental iron twice daily or 120 mg on alternate days for 6 weeks. In contrast to previous studies, 58% of participants in the twice daily group had a hemoglobin rise of at least 2 g/dL from baseline compared to 35.5% of participants in the alternate day group (*p* = 0.001). The mean rise of hemoglobin was also higher in the twice daily arm compared to alternate day arm (2.9 vs. 2.0 g/dL; *p* = 0.03). In our study, we compared a single daily dose of 60 mg to alternate day dose of 120 mg in 200 participants, a considerably larger sample size. The duration of supplementation was 2 months, more closely reflecting the real-life practice of rechecking hemoglobin in resource poor settings. The outcomes chosen were clinically meaningful including the mean change in hemoglobin, proportion of participants with a meaningful rise in hemoglobin, reticulocyte response and change in iron stores reflected by serum ferritin. Besides, participants in our study had more severe anemia (mean hemoglobin 6.53 g/dL and 6.68 g/dL in alternate day and consecutive day arms respectively) compared to those in the study by Kaundal et al.^12^.

While Kaundal et al.^12^ demonstrated that consecutive day administration with two doses is superior to alternate day single doses, our study provides new data on similar efficacy of a single daily dose compared to alternate day dosing in improving iron deficiency anemia.

We have no biochemical measurements to explain the disparity between results of our study and those of others that investigated effects on iron absorption. Yet, our results almost parallel the only other randomized controlled trial that used clinically important variables as primary outcomes^12^. While Mehta et al. reported significant increase in hemoglobin at 21 days among persons taking alternate day iron compared to daily iron, the primary outcome of their randomized controlled trial was hepcidin response^13^. Besides, the sample size was small (20 participants in each arm). Such instances of divergent results from studies using surrogate end points and clinical end points are not new and exemplify the need to perform rigorous randomized controlled trials on large number of participants with clinically meaningful endpoints and interventions closely reflecting real world experiences. Although the protocol for a randomized controlled trial comparing alternate day and daily iron has been published recently, it aims to recruit 52 patients and the primary outcome is the feasibility of recruiting the target sample size over 2 years^14^.

The major strengths of our study thus include: a large sample size, longer duration of iron supplementation, inclusion of participants with anemia rather than iron deficiency alone, use of commonly employed tolerable doses of iron and measurement of outcomes that are clinically meaningful. We also used double the dose of iron on alternate days ensuring that both groups received same total dose per unit time unlike previous studies. Lack of measurement of iron absorption studies or hepcidin levels could be considered limitations. Another limitation relates to the fact that inflammatory status of participants was not assessed at baseline or follow up using markers like C-reactive protein. Thus, the baseline ferritin as well as subsequent changes in ferritin levels could have been affected by inflammation; this might be a potential source of bias. Further, serum ferritin levels could be affected by recent iron intake; therefore, ferritin values could have acutely increased if the test was done within 1–3 days after the iron dose. Although this could have happened in both arms, it remains a potential limitation (Supplementary Dataset S1).

## Conclusion

This is the largest randomized controlled trial to our knowledge comparing alternate day versus daily administration of oral iron in iron deficiency anemia. Although, fractional iron absorption might be better with alternate day dosing, there is no difference in the clinically meaningful increase in hemoglobin between consecutive day single doses of oral iron compared to alternate day dosing. Further randomized controlled trials using different doses of iron, duration of administration and concurrent evaluation of iron kinetics may be needed to make recommendations on best practices regarding oral iron supplementation in iron deficiency anemia.

## Supplementary Information

Supplementary Information.

## Data Availability

All data generated or analyzed during this study are included in this published article and its supplementary information files (S1. Dataset).
