# Author Correction: Alternate day versus daily oral iron for treatment of iron deficiency anemia: a randomized controlled trial

**DOI:** 10.1038/s41598-023-37878-4

**Published:** 2023-07-03

**Authors:** Elamparithi Pasupathy, Ravichandran Kandasamy, Kurien Thomas, Aneesh Basheer

**Affiliations:** 1 Department of General Medicine, Sri Venkateshwaraa Medical College and Research Centre, Pondicherry, India; 2 Department of Biostatistics, Pondicherry Institute of Medical Sciences, Pondicherry, India; 3 Department of General Medicine, Pondicherry Institute of Medical Sciences, Pondicherry, India; 4 Department of General Medicine, DM Wayanad Institute of Medical Sciences, Naseera Nagar, Wayanad, Kerala 673577 India

Correction to: *Scientific Reports* 10.1038/s41598-023-29034-9, published online 01 February 2023

The Supplementary Information file published with this Article contained an error. The Supplementary Information file did not include information specifying what was the patient treatment allocation: the alternate day group or the daily dose group. The original Supplementary Information file is provided below.

This error has now been corrected in the Supplementary Information file that accompanies the original Article.

## Supplementary Information

Supplementary Information.

